# Antifungal Activity of the Essential Oil of *Illicium verum* Fruit and Its Main Component *trans*-Anethole

**DOI:** 10.3390/molecules15117558

**Published:** 2010-10-27

**Authors:** Yongfu Huang, Jianglin Zhao, Ligang Zhou, Jihua Wang, Youwen Gong, Xujun Chen, Zejian Guo, Qi Wang, Weibo Jiang

**Affiliations:** 1 College of Agronomy and Biotechnology, China Agricultural University, Beijing 100193, China; 2 College of Food Science and Nutritional Engineering, China Agricultural University, Beijing 100083, China

**Keywords:** Illiciaceae, *Illicium verum*, essential oil, *trans*-anethole, antifungal activity, plant pathogenic fungi

## Abstract

In order to identify natural products for plant disease control, the essential oil of star anise (*Illicium verum* Hook. f.) fruit was investigated for its antifungal activity on plant pathogenic fungi. The fruit essential oil obtained by hydro-distillation was analyzed for its chemical composition by gas chromatography (GC) and gas chromatography-mass spectrometry (GC-MS). *trans*-Anethole (89.5%), 2-(1-cyclopentenyl)-furan (0.9%) and *cis*-anethole (0.7%) were found to be the main components among 22 identified compounds, which accounted for 94.6% of the total oil. The antifungal activity of the oil and its main component *trans*-anethole against plant pathogenic fungi were determined. Both the essential oil and *trans*-anethole exhibited strong inhibitory effect against all test fungi indicating that most of the observed antifungal properties was due to the presence of *trans*-anethole in the oil, which could be developed as natural fungicides for plant disease control in fruit and vegetable preservation.

## 1. Introduction

Plant diseases are usually caused by plant pathogens including fungi, nematodes, bacteria, and viruses, among which fungi are the main pathogens, causing great yield losses in numerous important crops [1,2]. Over several decades, synthetic chemicals were always the chief means of preventing and controlling plant diseases, as they were effective, dependable and economic. However, abuse of synthetic agrochemicals has resulted in many problems, such as fungicide-resistance development of the pathogens [3], damage to the environment and human health, and ecosystem disruption [4]. So alternatives which are biodegradable, low-toxic and more effective, are in high demand. Research on natural products, which might substitute for synthetic fungicides or contribute to the development of new agents for plant disease control, has attracted much attention of investigators [5,6,7,8,9,10]. Plant essential oils are not only used as fragrance and flavouring agents in the food and beverages industries [11], but also may provide potential alternatives for use as plant fungal pathogenic control agents [12,13] as a large number of plant essential oils have been reported to have antifungal activities [14,15,16,17,18].

*Illicium verum* Hook. f. (Illiciaceae), a medium-sized evergreen plant named star anise, is mainly distributed in the tropical and subtropical zones of Asia. The fruits are frequently used as a well known spice in the food industry, and were also used for treatment of stomach aches and sepsis in eastern Asian traditional medicine [19,20]. The main component in *I. verum* essential oil was identified as *trans*-anethole [21]. The previous investigations suggested that *I. verum* essential oil had insecticidal, antimicrobial and antioxidative properties [21,22,23,24]. To the best of our knowledge, there have been no detailed studies on the use of *I. verum* essential oil and its main component *trans*-anethole against plant pathogenic fungi, besides a few reports on the antifungal activity screening of the oil [21,23]. We report here the antifungal activity of *I. verum* essential oil and *trans*-anethole against plant pathogenic fungi in order to provide additional data in supporting of their utilization and development as fungicides for plant disease control.

## 2. Results and Discussion

### 2.1. Essential oil analysis

Quantitative and qualitative analytical results of the fruit essential oil of *I. verum* by GC and GC-MS are shown in Table 1. Twenty-two compounds were identified, representing 94.6% of the whole composition of the oil. The most abundant component was *trans*-anethole (89.5%). Other main components included 2-(1-cyclopentenyl)-furan (0.9%) and *cis*-anethole (0.7%). Our result identifying the main component as *trans*-anethole is similar to the previous reports though other components were different from each other [17,21]. The reasons for this phenomenon may be different geographical environments, growth seasons and physiological age of the plant, in addition to the method of oil preparation [25,26].

**Table 1:** Chemical composition of the essential oil from the fruits of *I. verum*.

Compound^a^	RI^b^	RA (%)^c^
Camphene	950	0.2
β-Myrcene	991	0.1
α-Phellandrene	1003	0.1
δ-3-Carene	1016	0.3
*p*-Cymene	1029	0.1
Limonene	1033	0.4
*trans*-Ocimene	1040	0.1
γ-Terpinene	1060	0.1
Terpinolene	1100	0.1
Linalool	1114	0.3
Terpinen-4-ol	1177	0.2
Isobornyl thiocyanoacetate	1203	0.4
γ-Terpineol	1198	0.4
*cis*-Anethole	1254	0.7
*trans*-Anethole	1387	89.5
β-Elemene	1389	0.1
Cyperene	1398	0.2
β-Caryophyllene	1418	0.1
α-Caryophyllene	1450	0.1
(+)-9-Epiledene	1507	0.1
Cubebene	1672	0.1
2-(1-cyclopentenyl)-Furan	1694	0.9
Total identified		94.6
Monoterpene hydrocarbons		1.5
Oxygenated monoterpenes		1.3
Sesquiterpene hydrocarbons		0.4

^a^: The identified constituents are listed in their order of elution. ^b^: RI indicates the retention indices calculated against C_8_-C_40_ *n*-alkanes on the HP-5MS column. ^c^: RA indicates relative amount (peak area relative to the total peak area).

### 2.2. Antifungal activity by direct contact assay

The IC_50_ values of the oil and *trans*-anethole against mycelar growth of eleven plant pathogenic fungi are shown in Table 2. The essential oil had an obvious inhibitory activity against all test fungi, and the IC_50_ values ranged from 0.07 mg/mL to 0.25 mg/mL. This indicated that the oil had a broad spectrum of activity against all tested plant pathogenic fungi. Among them, *A. solani*, *B. maydis*, *F. graminearum*, *P. aphanidermatum* and *R. solani* were the relatively sensitive fungi with their IC_50_ values as 0.09 mg/mL, 0.07 mg/mL, 0.08 mg/mL, 0.09 mg/mL and 0.08 mg/mL, respectively. The positive control procymidone or carbendazim at 5 μg/mL completely inhibited the growth of all test fungi.

*trans*-Anethole, the dominant component in *I. verum* oil, has also been found in other plant essential oils [27,28,29]. It has been shown to possess insecticidal, larvicidal, and antimicrobial activities [30,31,32]. In order to compare the antifungal activity of *trans*-anethole with that of *I. verum* essential oil, *trans*-anethole was detected to display similar inhibitory activity on the test fungi with IC_50_ values closed to those of the oil (Table 2), which suggested that *trans*-anethole was a major contributor to the antifungal properties of *I. verum* essential oil.

**Table 2:** Antifungal activity (IC_50_) of *I. verum* fruit oil and *trans*-anethole against plant pathogenic fungi.

Fungus	Fruit oil IC_50_ (mg/mL)	*trans*-Anethole IC_50_ (mg/mL)
*Alternaria solani*	0.09	0.11
*Bipolaris maydis*	0.07	0.06
*Botryodiplodia theobromae*	0.11	0.09
*Fusarium graminearum*	0.08	0.10
*Fusarium oxysporum f. sp. cucumerinum*	0.16	0.17
*Fusarium oxysporum f. sp. lycopersici*	0.14	0.14
*Fusarium oxysporum* *f. sp. vasinfectum*	0.25	0.20
*Magnaporthe oryzae*	0.22	0.25
*Pythium aphanidermatum*	0.09	0.07
*Rhizoctonia cerealis*	0.10	0.11
*Rhizoctonia solani*	0.08	0.07

### 2.3. Antifungal activity by vapor contact assay

**Figure 1:**
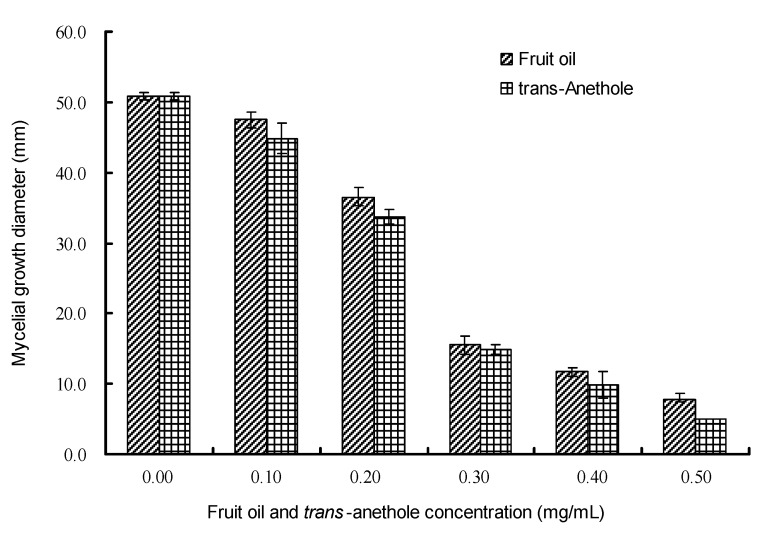
Effect of vapor components in *I. verum* fruit oil and *trans*-anethole on mycelial growth of *P. aphanidermatum* by vapor contact assay.

Two post-harvest pathogens, *P. aphanidermatum* and *B. theobromae*, were selected to evaluate the antifungal activity of the vapor components in *I. verum* essential oil as well as *trans*-anethole. Four days after exposure, the volatile components diffusing from the medium containing 0.5 mg/mL of *I. verum* essential oil inhibited the growth of *P. aphanidermatum* mycelia at a rate of 93.82%, and *trans*-anethole at the same concentration in medium more greatly inhibited the mycelia growth (Figure 1). Their IC_50_ values were determined as 0.23 mg/mL (*Y* = 4.386 *X* + 7.838, *R* = 0.989, where *Y* is the inhibitory probit value, *X* is concentration logarithm of the sample in the medium, and *R* is correlation coefficient) for the oil, and 0.20 mg/mL (*Y* = 4.045 *X* + 7.799, *R* = 0.983) for *trans*-anethole, respectively.

**Figure 2:**
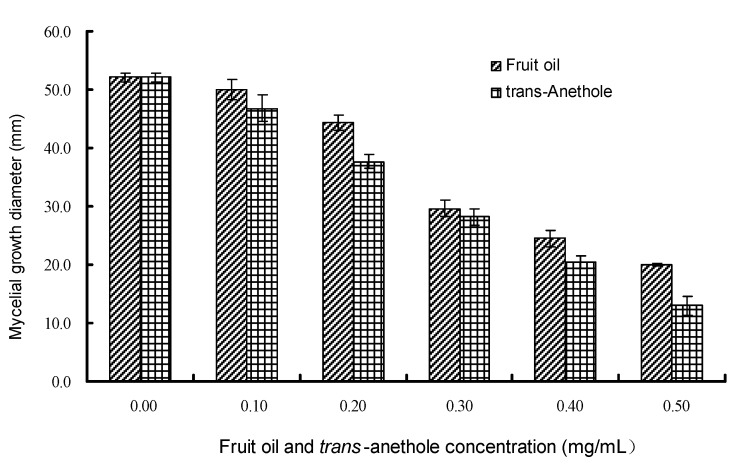
Effect of vapor components in *I. verum* fruit oil and *trans*-anethole on mycelial growth of *B. theobromae* by vapor contact assay.

Six days after exposure, both *I. verum* essential oil and *trans*-anethole at concentration of 0.5 mg/mL in medium inhibited the growth of *B. theobromae* mycelia at the rate of 68.20% and 83.04%, respectively (Figure 2). Their IC_50_ values were determined as 0.34 mg/mL (*Y* = 3.223 *X* + 6.479, *R* = 0.989) for the oil, and 0.27 mg/mL (*Y* = 2.972 *X* + 6.646, *R* = 0.979) for *trans*-anethole, respectively. Both the vapor components in *I. verum* essential oil and *trans*-anethole displayed a slightly stronger inhibitory effect on *P. aphanidermatum* (Figure 1) than on *B. theobromae* (Figure 2) which was similar to the results obtained from the direct contact assay (Table 2). At all concentrations in medium, *trans*-anethole displayed a very similar inhibitory rate to the essential oil against the test fungi, which suggested again that *trans*-anethole was the main active component among the volatiles in *I. verum* essential oil. The use of essential oil volatiles has several benefits over direct application of the oils themselves (*i.e.* reduced toxicity, ease of application). This indicates that the volatiles of *I. verum* essential oil could be used as fumigants for plant disease management such as post-harvest disease control in fruit and vegetable preservation.

### 2.4. Inhibitory activity on spore germination

**Figure 3:**
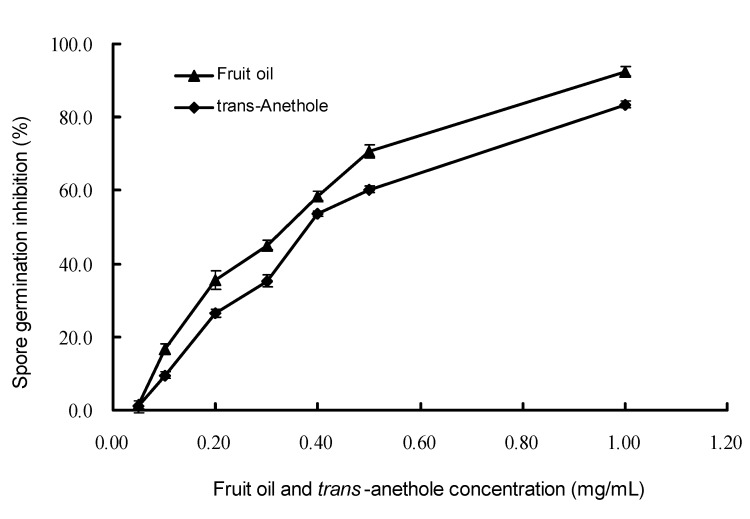
Inhibitory effect of *I. verum* fruit oil and *trans*-anethole on the spore germination of *M. oryzae*.

The inhibitory activity of the essential oil and *trans*-anethole on spore germination of *M. oryzae* was determined. The IC_50_ value of the oil against the spore germination was 0.32 mg/mL according to the regression equation (*Y* = 2.478 *X* + 6.202, *R* = 0.959, where *Y* is the inhibitory probit value, *X* is concentration logarithm of the essential oil in spore suspension, and *R* is correlation coefficient). The results obtained from the spore germination are shown in Figure 3. A negative control ran simultaneously in presence of acetone (5%, v/v) as that used in this study showed no inhibitory activity on spore germination, while the inhibitory effect of carbendazim (positive control) was 95.0% on spore germination at a concentration of 5.0 µg/mL. There was a strong inhibition on spore germination of *M. oryzae* at different concentrations of *I. verum* essential oil with 92.50% inhibition at 1.0 mg/mL of the oil, 70.50% inhibition at 0.5 mg/mL, and 58.40% at 0.4 mg/mL, respectively. *trans*-Anethole also displayed an obvious inhibition on spore germination of *M. oryzae*, but its activity was a little weaker than that of the essential oil. It gave an 83.50 % inhibition at 1.0 mg/mL, 60.23% inhibition at 0.5 mg/mL, and 53.50% inhibition at 0.4 mg/mL, respectively. It indicated that *trans*-anethole was the main component in the essential oil against spore germination of *M. oryzae*, though other minor components in the oil was also possible to contribute to the activity.

## 3. Experimental

### 3.1. Plant material

The dry fruits of *I. verum* were collected in September 2009 from Guangxi Province in the south of China, and were identified by Dr. Ye Wang of the Key Laboratory of Chemistry for Natural Products of Chinese Academy of Sciences in Guizhou Province of China. The voucher specimen (BSMPMI-200909001) was deposited in the Herbarium of the Institute of Chinese Medicinal Materials, China Agricultural University. The fruits were stored at 4 ºC before use.

### 3.2. Preparation of the essential oil

The fruits were broken into pieces, ground and then subjected to hydrodistillation in a Clevenger-type apparatus. The distilled oil was extracted with diethyl ether, and then dried over anhydrous sodium sulfate and preserved in a sealed dark glass vials at 4 ºC until required. Yield of the fruit essential oil was 7.48% (w/w at dry weight basis). *trans*-Anethole (purity min. 98.0%) was purchased from Tokyo Kasei Kogro Co., Ltd. (Japan).

### 3.3. Essential oil analysis

The composition of the oil was determined by the use of analytical GC (FID) and GC/MS technique. The same column and analysis conditions were used for both GC and GC/MS. The total neutral essential oil from *I. verum* fruits was analyzed by an Agilent 6890N Network GC (gas chromatograph) system with an Agilent 5973 Network mass selective detector. The machine was equipped with an HP-5MS (mass spectroscopy) column [30 m × 0.25 mm (5%-phenyl) -methylpolysiloxane capillary column, film thickness × 0.25 µm], a split-splitless injector at 250 ºC, and a flame ionization detector (FID) at 240 ºC. The oven temperature was programmed as follows: initial temperature 50 ºC for 1 min, increase 3 ºC /min up to 150 ºC, 1 min at 150 ºC, and then increase 8 ºC /min up to 230 ºC, 2 min at 230 ºC, finally, increase 20 ºC /min up to 260 ºC, 5 min at 260 ºC. The carrier gas was helium. The amount of sample injected was 1.0 µL (split ratio 1:20) and the ionization energy was 70 eV. The components were identified by comparison of their mass spectra with those of NIST 2002 library data of the GC-MS system, as well as by comparison of their retention indices (RI) with literature data [17,21,33,34,35,36,37,38,39]. The relative amounts (RA) of individual component of the essential oil was expressed as percentages of the peak area relative to the total peak area. RI value of each component was determined relatively to the retention times (RT) of a series of C_8_-C_40_ *n*-alkanes (Sigma) with linear interpolation on the HP-5MS column according to the Van den Dool approach [40].

### 3.4. Test fungi

Eleven plant pathogenic fungi, namely *Alternaria solani*, *Bipolaris maydis*, *Botryodiplodia theobromae*, *Fusarium graminearum*, *Fusarium oxysporum* f. sp. *cucumerinum*, *Fusarium oxysporum* f. sp. *lycopersici*, *Fusarium oxysporum* f. sp. *vasinfectum*, *Magnaporthe oryzae*, *Pythium aphanidermatum*, *Rhizoctonia cerealis*, and *Rhizoctonia solani* were kindly provided by the Department of Plant Pathology of China Agricultural University. They were maintained on potato dextrose agar (PDA) medium and then stored at 4 ºC. All the fungi were activated and then subcultured from 7 to 14 days in darkness at 25 ± 2 °C by transferring from the stock cultures to PDA medium in Petri dishes before use.

### 3.5. Evaluation of the antifungal activity

#### 3.5.1. Determination of antifungal activity by direct contact assay

Antifungal activity was studied by using an *in vitro* direct contact assay which was also called mycelial radial growth inhibition assay [41,42,43,44]. Briefly, the test sample (essential oil or *trans*-anethole) was mixed with PDA medium at 50 ºC to obtain its final concentrations as 0.050 mg/mL, 0.080 mg/mL, 0.10 mg/mL, 0.15 mg/mL, 0.20 mg/mL, 0.25 mg/mL and 0.30 mg/mL, respectively. Then 10 mL of PDA medium was poured into a 6-cm diameter Petri dish before solidification. Discs (5-mm diameter) of the mycelial plugs from the edge of cultured fungal colony were cut and placed mycelial surface down on the centre of dishes. Three replicates were used for each treatment. All plates were then cultivated in the dark at 25 ± 2 ºC. Mycelial diameter was measured when the hyphae of the control extended to the edge of dishes. The dishes without sample in PDA medium were used as the negative controls. Procymidone (Aldrich) at 5.0 μg/mL in medium was used as positive control for *P. aphanidermatum* test, and carbendazim (Aldrich) at 5.0 μg/mL in medium was used as positive control for the other fungal test. The percentage (%) of mycelial growth inhibition was determined as [(*M*_c_ – *M*_t_)/*M*_c_] ×100, where *M*_c_ is an average of three replicates of mycelial growth diameter (mm) increase of the negative controls, and *M*_t_ is an average of three replicates of mycelial growth diameter (mm) increase of plates treated with the essential oil. The median inhibitory concentration (IC_50_) against test fungi was calculated using the linear relation between the inhibitory probability and concentration logarithm according to methods outlined by Finney [45].

#### 3.5.2. Determination of antifungal activity by vapor contact assay

The effects of either volatile components in the oil or *trans*-anethole were tested using 15-cm diameter hermetical glass plate which contained two compartment 6-cm diameter Petri dishes, one contained PDA inoculated with either *P. aphanidermatum* or *B. theobromae*, and another contained 10 mL of PDA containing essential oil or *trans*-anethole at various concentrations of 0.20 mg/mL, 0.50 mg/mL, 1.0 mg/mL, 2.0 mg/mL and 5.0 mg/mL. PDA plate treated without the oil was used as the control. In this way, the fungi were not in direct contact with the sample and any effect on growth of the mycelia could be attributed to the volatile components. Three replicates were used for each treatment. All plates were then cultivated in the dark at 25 ± 2 ºC for 4 days for *P. aphanidermatum* or 6 days for *B. theobromae*. Mycelia diameter was measured when the diameter of the hyphae extended to the sides of dishes [44,46]. Mean growth measurements were calculated from three replicates of each test fungus. The percentage (%) of the mycelia growth inhibition and IC_50_ values of the sample against the test fungi were calculated referring to the paragraph mentioned above.

#### 3.5.3. Determination of inhibitory activity on spore germination

Rice blast fungus, *Magnaporthe oryzae* (strain P131) was maintained on oatmeal-tomato agar medium (oatmeal 30 g/L, tomato juice 150 mL/L, and agar 20 g/L) at 25 ºC. The spores were prepared from 7-day-old cultures of *M. oryzae*, according to our previous reports [47,48]. The test sample (essential oil or *trans*-anethole) dissolved in 10 % (v/v) acetone solution with its different concentrations was mixed with equivalent volume of fungal spore suspension containing 2 × 10^6^ spores per mL. The mixture was then placed on separate concave glass slides. The final sample concentrations were 0.050 mg/mL, 0.10 mg/mL, 0.20 mg/mL, 0.30 mg/mL, 0.40 mg/mL, 0.50 mg/mL and 1.0 mg/mL, respectively containing 5 % (v/v) acetone, together with two controls, *i.e.* 5 % (v/v) acetone as the negative control, and 5.0 µg/mL carbendazim as the positive control. Three replicates were used for each treatment. The slides containing spores were incubated in a moist chamber at 25 ± 2 ºC for 7 h. Each slide was then observed under the microscope for spore germination status. About 300 spores were observed to detect spore germination. The percentage (%) of the spore germination inhibition was determined as [(*G*_c_ – *G*_t_)/*G*_c_] ×100, where *G*_c_ is an average of three replicates of germinated spore numbers in the negative control, and *G*_t_ is an average of three replicates of germinated numbers in the treated sets. The IC_50_ calculation for the spore germination inhibition was the same as that for mycelial growth inhibition described above.

## 4. Conclusions

This study showed that the fruit essential oil of *I. verum* and *trans*-anethole had a wide inhibitory spectrum of activity against plant pathogenic fungi. The antifungal activity of the oil can be attributed by its high content of *trans*-anethole, which was confirmed as the main active component among the volatile compounds in the oil. Both the fruit essential oil and *trans*-anethole from *I. verum* could be developed as the natural fungicides (*i.e.* fumigants) for plant disease control in fruit and vegetable preservation.
